# Elevated Serum Levels of Acid Sphingomyelinase in Female Patients with Episodic and Chronic Migraine

**DOI:** 10.3390/antiox14020159

**Published:** 2025-01-29

**Authors:** Alberto Ouro, Mónica Castro-Mosquera, Mariña Rodríguez-Arrizabalaga, Manuel Debasa-Mouce, Antía Custodia, Marta Aramburu-Núñez, Daniel Romaus-Sanjurjo, Josefina Casas, Isabel Lema, José Castillo, Rogelio Leira, Tomás Sobrino

**Affiliations:** 1 NeuroAging Group Laboratory (NEURAL), Clinical Neurosciences Research Laboratory (LINC), Health Research Institute of Santiago de Compostela (IDIS), 15706 Santiago de Compostela, Spain; monicacastro.mosquera@usc.es (M.C.-M.); marina.rodriguez.arrizabalaga@sergas.es (M.R.-A.); manuel.debasa@rai.usc.es (M.D.-M.); antia.custodia.malvido@sergas.es (A.C.); marta.aramburu.nunez@sergas.es (M.A.-N.); daniel.romaus.sanjurjo@sergas.es (D.R.-S.); 2 Centro de Investigación Biomédica en Red de Enfermedades Neurodegenerativas, Instituto de Salud Carlos III, 28029 Madrid, Spain; 3 Research Unit on BioActive Molecules, Department of Biological Chemistry, Institute for Advanced Chemistry of Catalonia (IQAC-CSIC), 08034 Barcelona, Spain; fina.casas@iqac.csic.es; 4 Liver and Digestive Diseases Networking Biomedical Research Centre (CIBEREHD), Instituto de Salud Carlos III, 28029 Madrid, Spain; 5 Corneal Neurodegeneration Group (RENOIR), Clinical Neurosciences Research Laboratory (LINC), Health Research Institute of Santiago de Compostela (IDIS), 15706 Santiago de Compostela, Spain; isabel.lema.gesto@sergas.es; 6 Department of Surgery and Medical-Surgical Specialties, Faculty of Optics and Optometry, Universidade de Santiago de Compostela, 15706 Santiago de Compostela, Spain; 7 Instituto Galego de Oftalmoloxía (INGO), Hospital Provincial de Conxo, 15706 Santiago de Compostela, Spain; 8 Neuroimaging and Biotechnology Laboratory (NOBEL), Clinical Neurosciences Research Laboratory (LINC), Health Research Institute of Santiago de Compostela (IDIS), 15706 Santiago de Compostela, Spain; jose.castillo.sanchez@sergas.es; 9 Department of Neurology, Hospital Clínico Universitario, Universidad de Santiago de Compostela, 15705 Santiago de Compostela, Spain; rogelio.leira.muino@sergas.es

**Keywords:** acid sphingomyelinase, endothelial dysfunction, inflammation, migraine, oxidative stress, serum, sphingomyelin, vascular

## Abstract

Migraine is one of the most common neurological disorders and the second most disabling human condition. The molecular mechanisms of migraine have been linked to neuropeptide release, endothelial dysfunction, oxidative stress and inflammatory processes. Acid sphingomyelinase (aSMase) is a secreted enzyme that leads to sphingomyelin degradation to produce ceramide. Its activity has been associated with several molecular processes involved in migraine. Therefore, this cross-sectional study aims to study the potential role of aSMase in patients with episodic and chronic migraine. In this cross-sectional pilot study, serum samples from female healthy controls (n = 23), episodic migraine (EM) patients (n = 31), and chronic migraine (CM) patients (n = 28) were studied. The total serum levels of aSMase were determined by ELISA. In addition, the serum levels of sphingomyelin (SM), dihydro-sphingomyelin (dhSM), ceramide (Cer), and dihydro-ceramide (dhCer) were determined by mass spectrometry as biomarkers involved in the main molecular pathways associated with aSMase. aSMase serum levels were found significantly elevated in both EM (3.62 ± 1.25 ng/mL) and CM (3.07 ± 0.95 ng/mL) compared with controls (1.58 ± 0.72 ng/mL) (*p* < 0.0001). ROC analysis showed an area under the curve (AUC) of 0.94 (95% CI: 0.89–0.99, *p* < 0.0001) and 0.90 (95% CI: 0.81–0.99, *p* < 0.0001) for EM and CM compared to controls, respectively. Regarding other biomarkers associated with aSMase’s pathways, total SM serum levels were significantly decreased in both EM (173,534 ± 39,096 pmol/mL, *p* < 0.01) and CM (158,459 ± 40,010 pmol/mL, *p* < 0.0001) compared to the control subjects (219,721 ± 36,950 pmol/mL). Elevated serum levels of aSMase were found in EM and CM patients compared to the control subjects. The decreased SM levels found in both EM and CM indicate that aSMase activity plays a role in migraine. Therefore, aSMase may constitute a new therapeutic target in migraine that should be further investigated.

## 1. Introduction

Migraine is considered a complex neurovascular and neuroinflammatory brain disorder, including both vascular and neural mechanisms, that affects over 1 billion individuals across the world [1]. Consequently, migraine is recognized as one of the main causes of disability under the age of 50, with women as the main affected, with an estimation of a 3:1 ratio [2].

Two types of migraine, episodic migraine (EM) and chronic migraine (CM), are mainly differentiated by the frequency of crises following the International Headache Society (IHS) classification [3]. In this regard, CM is characterized by headache episodes occurring on 15 or more days per month for more than 3 months, with the features of migraine headache on at least 8 days per month.

Although the molecular mechanisms of migraine remain unclear, inflammation and oxidative stress seem to be involved according to data from experimental models and plasma biomarkers from patients [4,5,6,7]. In addition, nociceptive inputs from the raphe and locus coeruleus nuclei [8], trigeminovascular (TGV) system activation, and the cortical spreading depression (CSD) phenomenon [9] are also involved in migraine onset. Moreover, secreted inflammatory vasoactive peptides, such as Calcitonin-gene related peptide (CGRP), promote dilatation of the meningeal vessels, induce oxidative stress [10], modulate endothelial function [11,12], and could trigger blood–brain barrier (BBB) disruption during migraine attacks [13].

Furthermore, oxidative stress is a key factor for the development of endothelial dysfunction [14], which is involved in migraine pathophysiology [7,10,15,16,17,18]. In this regard, SM has been described to have dual antioxidant/pro-oxidant properties [19]. Sphingolipids have been described as pleiotropic molecules involved in cell regulation of several functions [20]. Sphingolipids regulate multiple pathways implicated in the control of cell functions like proliferation, apoptosis, autophagy, inflammation, endothelial homeostasis and angiogenesis, among others [21,22,23,24,25]. The dysregulation of sphingolipid metabolism leads to the loss of cellular and organism homeostasis. Consequently, sphingolipids have been described to be involved in different diseases such as cancer, diabetes, neurodegeneration and cardiovascular diseases [26,27,28,29]. Sphingomyelin (SM) is a dominant sphingolipid in membranes of mammalian cells. It is considered a key molecule of sphingolipid metabolism since its enzymatic breakdown by Sphingomyelinases (SMases) leads to an increase in ceramide (Cer) concentration. In this regard, it has been widely reported that the SM-Cer axis is associated with several diseases, such as atherosclerosis, diabetes, neurodegeneration, stroke, dementia, oxidative stress, and systemic inflammation, among others [27,28,29,30,31,32,33,34,35,36,37]. Interestingly, changes in bioactive lipids, such as lysophospholipids and sphingolipids, have been determined [38,39,40,41] in the plasma of patients with migraine, as well as transcriptomic alterations in lipid metabolism in migraine models and patients with migraine [42,43]. In addition, variations in SM and Cer levels in patients with migraine have been determined by different groups [39,40]. Specifically, a previous study demonstrated that elevated SM levels were associated with a higher probability of migraine attacks in patients with EM [40].

Considering all the aforementioned evidence, and the putative relationship between a secreted form of SMase, known as acid SMase (aSMase), leading to an increase in SM levels, and migraine, our study aims to elucidate the potential implication of aSMase in migraine to confirm the hypothesis that aSMase serum levels are elevated in EM and CM patients compared to healthy subjects, as well as the levels of SM. For this purpose, we designed a cross-sectional pilot study of a cohort of healthy controls, EM and CM patients in order to study the association of serum levels of aSMase and their metabolites with migraine.

## 2. Materials and Methods

### 2.1. Subjects

All patients and control subjects were prospectively recruited from the Headache Unit of the Neurology Department at Hospital Clínico Universitario of Santiago de Compostela. The patients with migraine were classified according to the International Classification of Headache Disorders, 3rd edition criteria [3].

### 2.2. Clinical Study Protocol

This cross-sectional study was conducted following the Declaration of Helsinki of the World Medical Association (2008) and approved by the Ethics Committee of the Servizo Galego de Saúde (2016/079). Written informed consent was obtained from each subject included in this study. All subjects were older than 18 years. For the molecular determinations, venous blood samples were collected in a fasting period and avoiding periods of menstruation with anticoagulant-free Vacutainer tubes (Becton Dickinson, San Jose, CA, USA) during a headache-free period of 24 h before the visit. The subjects had not previously consumed medication during this headache-free period.

The exclusion criteria were the following: (1) chronic inflammatory conditions; (2) severe systemic diseases; (3) neuroendocrine tumors; (4) multisystemic trauma; (5) other neurological diseases; (6) vascular risk factors (arterial hypertension, diabetes mellitus, coronary disease, smoking, dyslipidemia and obesity with BMI ≧ 35 kg/m^2^; (7) excessive sports activity (vigorous physical activity more than 5–6 days a week, 7–10 h of vigorous exercise a week, or vigorous exercise for more than 2–3 h daily); (8) pregnancy or lactation; and 9) other forms of chronic headache.

### 2.3. Laboratory Tests

For the determination of serum levels of biomarkers, 4.5 mL of blood from the antecubital vein was collected during fasting. Samples were obtained during a pain-free interval of 24 h and after an overnight fast. All samples were kept in chemistry test Vacutainer anticoagulant-free tubes (Becton Dickinson, San Jose, CA, USA) and centrifuged at 1700× *g* for 15 min. The serum was immediately frozen and stored at −80 °C for further analysis.

Serum levels of human aSMase were determined with commercial ELISA kits following the manufacturer’s instructions (Abcam, Cambridge, UK, Ref: ab277075), with a sensibility of 17.33 pg/mL and intra-assay coefficient of variation of 4.9 pg/mL. To determine the concentrations by ELISA, an extrapolation was performed with the internal standards of the kit. The determinations were performed in a laboratory blinded to clinical data.

### 2.4. Sphingolipidomics

Lipids were extracted and determined from serum samples as described in [44]. A total of 750 µL of a methanol/chloroform (2:1, *v*/*v*) solution containing internal standards (N-dodecanoylsphingosine, N-dodecanoylglucosylsphingosine, N-dodecanoylsphingosylphosphorylcholine, C17-dihydrosphingosine and C17-dihydrosphingosine-1-phosphate, 0.2 nmol each, from Avanti Polar Lipids) was added to 75 µL of serum. The samples were extracted at 48 °C overnight and cooled. Then, 75 µL of 1 M KOH in methanol was added, and the mixture was incubated for 2 h at 37 °C. Following the addition of 75 µL of 1 M acetic acid, the samples were evaporated to dryness and stored at −80 °C until the analysis. Before the analysis, 150 µL of methanol was added to the samples, centrifuged at 13,000× *g* for 5 min, and 130 µL of the supernatant were transferred to a new vial and injected.

Lipids were analyzed by liquid chromatography–high-resolution mass spectrometry (LC-HRMS). LC-HRMS analysis was performed using an Acquity ultra-high-performance liquid chromatography (UHPLC) system (Waters, Milford, MA, USA) connected to a Time of Flight (LCT Premier XE) Detector. Full-scan spectra from 50 to 1800 Da were acquired, and individual spectra were summed to produce data points each of 0.2 sec. Mass accuracy at a resolving power of 10,000 and reproducibility were maintained by using an independent reference spray via the LockSpray interference. The capillary voltage was set to 3.0 kV, the desolvation temperature was set to 350 °C, and the desolvation gas flow was set to 600 L/h. Lipid extracts were injected onto an Acquity UHPLC BEH C8 column (1.7 µm particle size, 100 mm × 2.1 mm, Waters, Cork, Ireland) at a flow rate of 0.3 mL/min and a column temperature of 30 °C. The mobile phases were methanol with 2 mM ammonium formate and 0.2% formic acid (A)/water with 2 mM ammonium formate and 0.2% formic acid (B). A linear gradient was programmed as follows: 0.0 min: 20% B; 3 min: 10% B; 6 min: 10% B; 15 min: 1% B; 18 min: 1% B; 20 min: 20% B; 22 min: 20% B. Positive identification of compounds was based on the accurate mass measurement with an error <5 ppm and its LC retention time compared with that of an authentic standard (92%). Quantification was carried out using the extracted ion chromatogram of each compound using 50 mDa windows. The relative peak area for internal standards and available standards at different concentrations was checked every day. The linear dynamic range was determined by injecting mixtures of internal and natural standards. Following, natural standards were used. For Cer standards, we used N-palmitoyl-sphingosine, N-stearoyl-sphingosine, N-lignoceroyl-sphingosine and N-nervonoyl-sphingosine. DHcer standards used were N-palmitoyl-dihydrosphingosine, N-stearoyl-dihydrosphingosine, N-lignoceroyldihydrosphingosine and N-nervonoyl-dihydrosphingosine. The SM standards used were N-palmitoylsphingosylphosphorylcholine, egg SMs (predominant C16:0SM) and brain SMs (C18:0SM, C24:0SM and C24:1SM in known percentages). The glucosylceramide standard used was N-palmitoylglucosylsphingosine. The lactosylceramide standard used was N-palmitoyl-lactosylsphingosine. Since the standards for all identified lipids were not available, the amounts of lipids are given as pmol equivalents relative to each specific standard (Figure S1).

### 2.5. Data and Statistical Analysis

The results are expressed as percentages for categorical variables, mean or median and interquartile range for continuous variables, depending on the normal or not-normal distribution of data. Normality was determined by the Kolmogorov–Smirnov test. Two-tailed statistical test post hoc ANOVA-Tukey was used for the normally distributed discrete/continuous variables in more than two groups.

The sensitivity and specificity of the different biomarkers were represented graphically by receiver operating characteristic (ROC) curves. A value of *p* < 0.05 was considered significant. The cut-offs were determined by Youden’s index. Statistical analysis was performed using SPSS version 27.0 (IBM Corp., Armonk, NY, USA) and GraphPad Prism 10.0 (GraphPad Software, Inc., San Diego, CA, USA). There were no missing data.

Since this is a secondary analysis from cohorts of previous works [45,46], no formal sample size calculation was performed. However, a post hoc power analysis based on the obtained results from the present study and using our primary outcome (i.e., aSMase concentrations) confirmed a 95% power to detect a 0.5 ng/mL difference in aSMase levels between the study groups (migraine patients vs. controls), with an SD of 0.05. These statistical power analyses were performed with GraphPad Prism 10.0 (GraphPad Software, Inc., San Diego, CA, USA).

## 3. Results

### 3.1. Elevated Acid Sphingomyelinase Concentrations in Patients with Migraine

**Figure 1:**
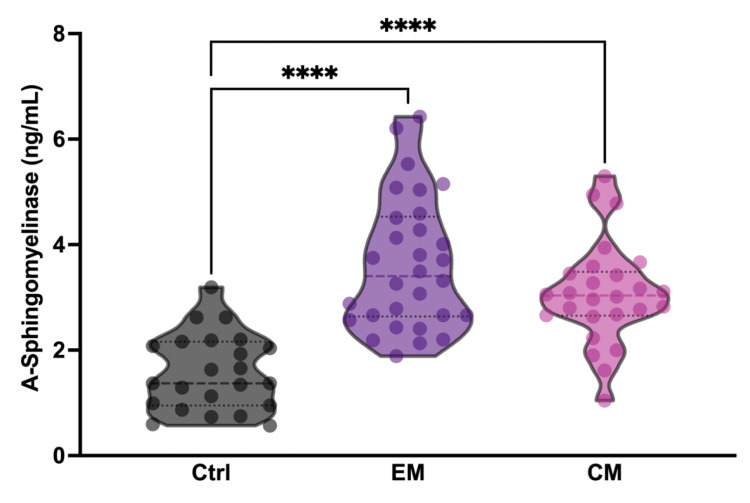
Acid sphingomyelinase (aSMase) serum levels are increased in patients with migraine compared to controls. Analysis of aSMase levels in serum samples from healthy patients (Ctrl), patients with episodic migraine (EM), and patients with chronic migraine (CM). The results are presented by a violin graph and expressed as the mean and interquartile. The statistics were analyzed by one-way ANOVA test (**** *p* < 0.0001).

To determine whether serum aSMase levels in patients with migraine increased between attack periods, three different analysis groups were established: healthy subjects (Ctrl), patients with episodic migraine (EM), and patients with chronic migraine (CM). The groups were matched for age and sex. Specifically, all study subjects were female (Table 1). The aSMase levels were significantly elevated in both EM (3.62 ± 1.25 ng/mL,) and CM (3.07 ± 0.95 ng/mL,) patients with migraine compared to the controls (1.58 ± 0.72 ng/mL) (*p* < 0.0001); meanwhile, no differences were found between EM and CM (*p* = 0.1159) (Figure 1; Table 1). These data demonstrate that basal aSMase concentrations are elevated in patients with migraine between attack-free periods.

#### ROC Curves Reveal That Serum Concentration of aSMase Is a Good Biomarker for Both Episodic and Chronic Migraine

**Figure 2:**
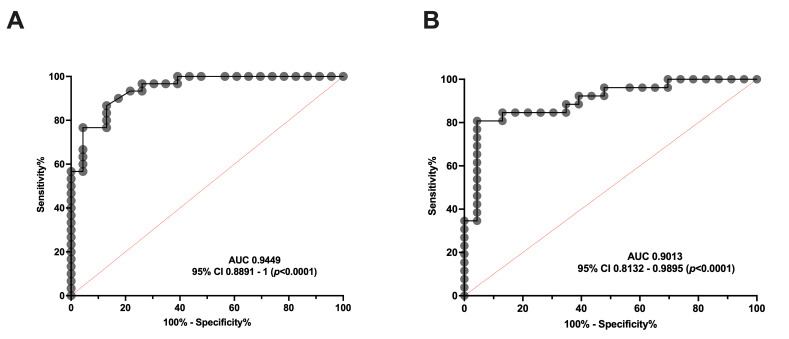
ROC curve analysis comparing aSMase serum levels in healthy subjects and patients with migraine. (**A**) ROC curve analysis comparing aSMase levels of control vs. episodic migraine patients (EM). (**B**) aSMase levels ROC curve analysis of control vs. chronic migraine patients (CM).

To determine whether serum aSMase levels are capable of discriminating between healthy subjects and patients with migraine, an ROC curve analysis was performed to determine the specificity and sensitivity of the serum levels of this enzyme in the EM and CM groups. As can be observed in Figure 2, the aSMase levels clearly distinguish EM (Figure 2A) and CM (Figure 2B) patients from the control subjects. Specifically, the ROC analysis showed an area under the curve (AUC) of 0.94 (95% CI: 0.89–0.99, *p* < 0.0001) and 0.90 (95% CI: 0.81–0.99, *p* < 0.0001) for EM and CM compared to the controls, respectively. Furthermore, aSMase levels ≥ 2.0 ng/mL identify patients with CM versus controls with a sensitivity of 87% and a specificity of 87%, whereas aSMase levels ≥ 2.4 ng/mL identify patients with EM versus controls with a sensitivity of 81% and a specificity of 87%.

Consequently, our data indicate that serum aSMase levels are significantly more elevated in migraine than healthy subjects, being a good biomarker for migraine, but not for discriminating between EM and CM.

### 3.2. Sphingolipidomics Demonstrated a Significant Reduction in Serum Sphingomyelin in Patients with Migraine

**Figure 3:**
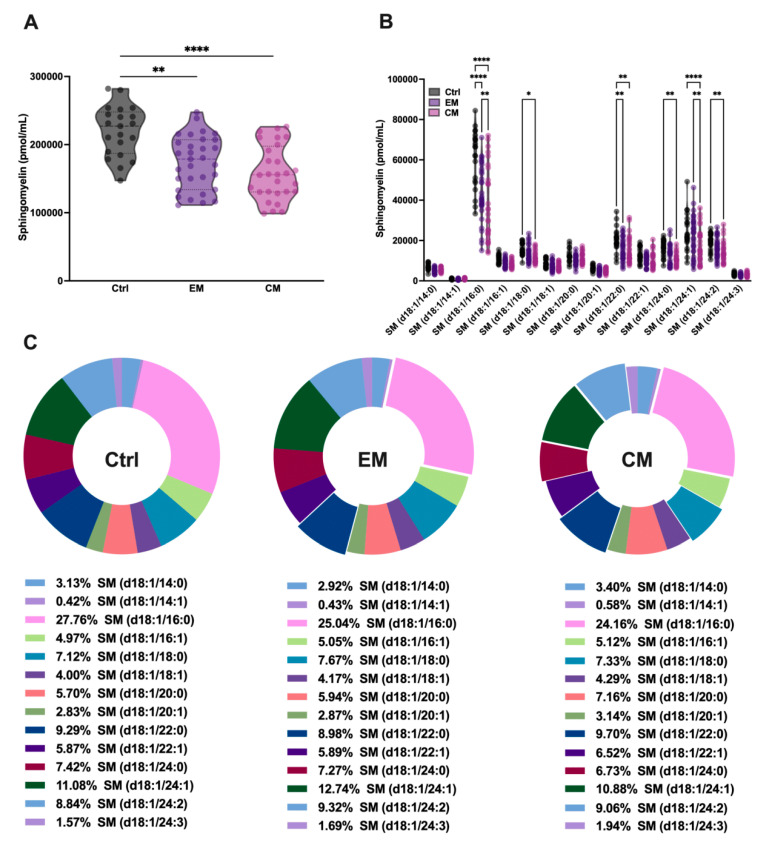
Sphingolipidomics showed a great reduction in sphingomyelin (SM) levels, which indicated high aSMase activity in the serum of patients with migraine. (**A**) Representation in pmol/mL of total SM serum levels by groups. The results are presented as mean and interquartile and analyzed by one-way ANOVA (** *p* < 0.001, **** *p* < 0.0001) compared to the control group. (**B**) Representation in pmol/mL of the serum levels of SM species. The results are expressed as the mean and interquartile, and analyzed by one-way ANOVA (* *p* < 0.05, ** *p* < 0.001, **** *p* < 0.0001) compared to the control group. (**C**) Representation in percentage (%) of the serum levels of SM species. The results are presented as a pie chart. The representative fragments of each species that stand out indicate significant changes concerning the control but not in the % of the total, as indicated in Figure 3B.

It is well known that SMase binds to SM with high affinity in order to catalyze its hydrolysis and produce ceramide. To determine whether the increased serum concentration of aSMase correlates with a decrease in its natural substrate SM, serum SM levels were determined by sphingolipidomics. As expected, the total SM levels were significantly decreased in both EM (173.53 ± 39.09 pmol/mL, *p* < 0.01) and CM (158.46 ± 40.01 pmol/mL, *p* < 0.0001) compared to the control subjects (219.72 ± 36.95 pmol/mL) (Figure 3A; Table 2).

Furthermore, when analyzing all the SM species that differ in the length of the fatty acid chain, it was observed that all the major SM species in serum were affected by aSMase activity, showing significant reductions in both EM and CM compared to the controls (Figure 3B; Table 2). However, as might be expected given the reduction in all SM species, the overall proportion of these species was not affected in the serum profile (Figure 3C; Table 2).

#### 3.2.1. ROC Curves Demonstrated That Serum SM Levels Are a Good Biomarker for Both Episodic and Chronic Migraine

**Figure 4:**
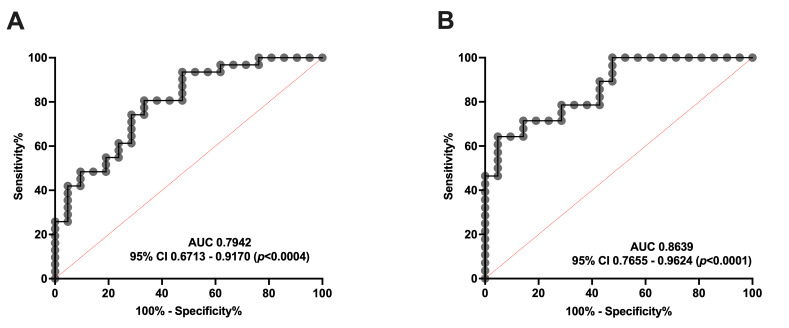
ROC curve analysis comparing SM concentration in healthy subjects vs. patients with migraine (**A**) ROC curve analysis comparing SM levels of controls with episodic migraine patients (EM). (**B**) SM levels ROC curve analysis of controls versus chronic migraine patients (CM).

Additionally, we performed an ROC curve analysis to clarify whether serum SM levels are able to distinguish between healthy subjects and patients with migraine. As shown in Figure 4, SM levels can discriminate EM (Figure 4A) and CM (Figure 4B) from controls. Specifically, the ROC analysis showed an AUC of 0.79 (95% CI: 0.67–0.92, *p* = 0.0004) and 0.86 (95% CI: 0.76–0.96, *p* < 0.0001) for EM and CM compared to the controls, respectively. Furthermore, SM levels ≥ 203.04 pmol/mL identify EM versus controls with a sensitivity of 74% and a specificity of 71%. Meanwhile, SM levels ≥ 177.25 pmol/mL identify patients with CM versus controls with a sensitivity of 71%, and a specificity of 86%.

Taking these data into account, serum SM levels may be considered a good biomarker, and a potential target, for migraine. Interestingly, no differences were found between EM and CM.

#### 3.2.2. Increased Serum aSMase Levels Selectively Degrade the Major dhSM Species 18:0/16:0 in Patients with Migraine

**Figure 5:**
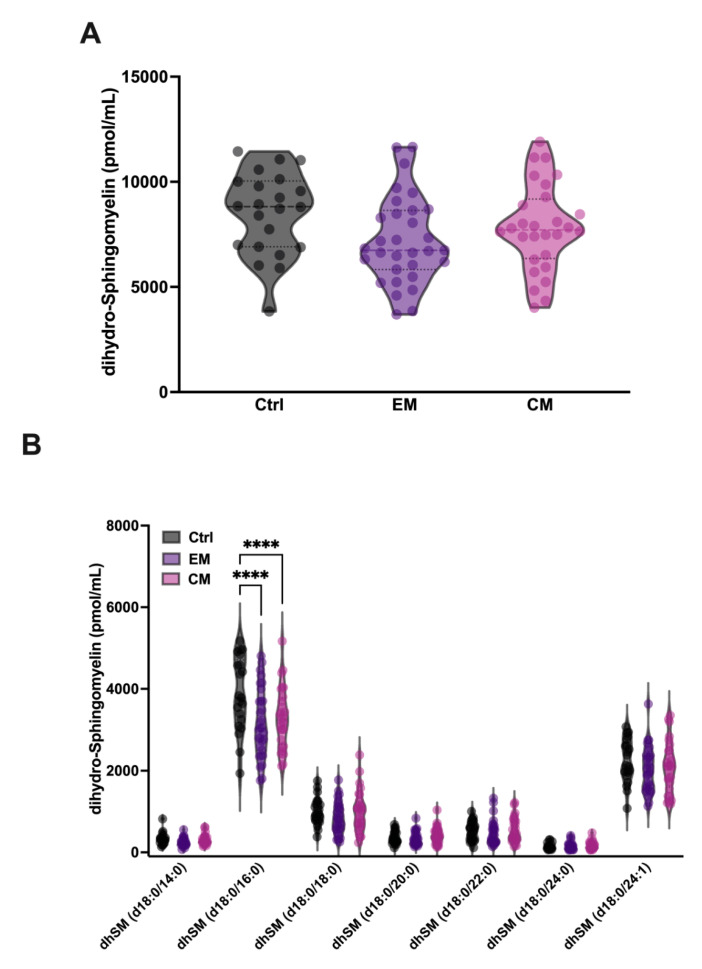
Sphingolipidomics of dihydro-sphingomyelin (dhSM) in the serum of patients with migraine. (**A**) Representation in pmol/mL of the total serum dhSM levels. The results are presented as mean and interquartile and analyzed by one-way ANOVA compared to the control group. (**B**) Representation in pmol/mL of the serum levels of dhSM species. The results are expressed as the mean and interquartile, and analyzed by one-way ANOVA (**** *p* < 0.0001) compared to the control group.

In addition, it has been described that aSMase is able to degrade the SM analogue dihydrosphingomyelin (dhSM) with lower affinity. dhSM differs from SM in the absence of the double bond at the 4–5 position of the sphingoid base and is found in mammalian cells and serum in a minority concentration compared to SM. Therefore, the levels of dhSM in serum were also determined. Our results show that although the levels of dhSM are not significantly affected in EM and CM compared to controls (Figure 5A, Table 2), a significant decrease in the levels of its major species dhSM 18:0/16:0 was observed both in EM (3160.3 ± 838 pmol/mL; *p* < 0.0001) and CM (3284.1 ± 738.8 pmol/mL; *p* < 0.0001) compared to the control group (3872.2 ± 909 pmol/mL) (Figure 5B, Table 2). These data could be explained by the low affinity of aSMase against dhSM, and the requirement of higher concentrations of substrate for the onset of its activity.

### 3.3. No Alterations in Serum Ceramide Were Detected in Patients with Migraine Compared with Healthy Subjects

**Figure 6:**
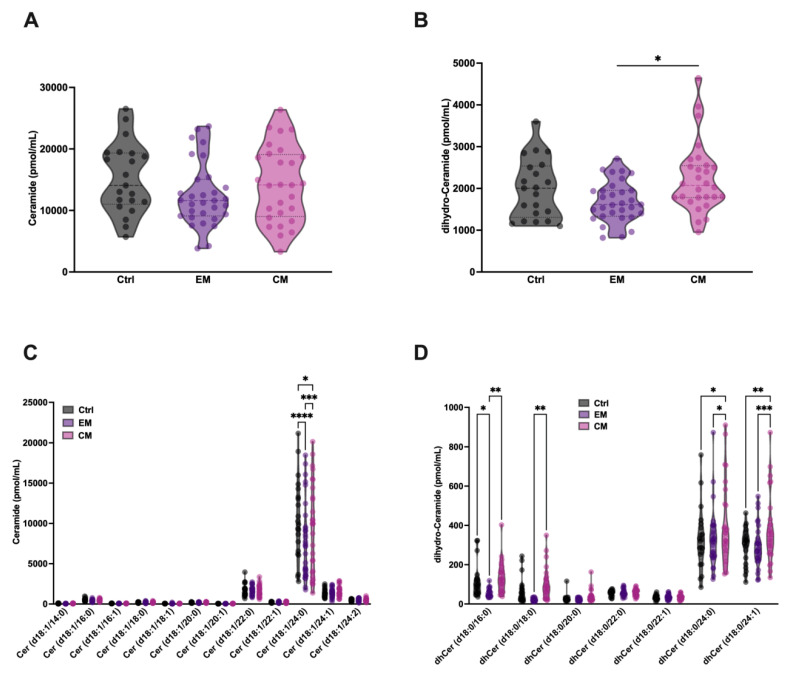
Total serum levels of ceramide (Cer) and dihydro-ceramide (dhCer) were not significantly different in patients with migraine compared with control subjects. Representation in pmol/mL of the serum levels of total Cer (**A**) and dhCer (**B**). The results are presented as mean and interquartile and analyzed by one-way ANOVA compared to the control group; Representation in pmol/mL of the levels of Cer (**C**) and dhCer (**D**) species. The results are expressed as the mean and interquartile, and analyzed by one-way ANOVA (* *p* < 0.05, ** *p* < 0.001, *** *p* < 0.0005, **** *p* < 0.0001) compared to the control group.

Given the increased degradation of SM and dhSM by aSMase in serum, an increase in the levels of ceramide (Cer) and its analogue dihydroceramide (dhCer) in serum would be expected. Based on this, their levels were determined in the different groups. Surprisingly, no increases in the levels of Cer or dhCer were detected in both EM and CM; however, a significant elevation in dhCer in serum was observed in CM compared with EM (Figure 6 A,B). dhCer differs from Cer in the absence of the double bond at the 4–5 position of the sphingoid base of the fatty acid chain.

Furthermore, a small but significant reduction in Cer 18:1/24:0 levels was observed in both groups compared to healthy subjects (Figure 6C). This reduction is also observed in different dhCer species (Figure 6D), being more obvious in patients with EM than in those with CM, where a significant increase in dhCer levels is observed in CM compared to EM. These results are in accordance with a previous study with a small cohort of EM female patients, compared with healthy controls, where a significant decrease in total Cer and dhCer was detected [40]. On the other hand, an increase in dhCer species 18:0/24:0 and 18:0/24:1 was observed in patients with CM compared to healthy subjects.

## 4. Discussion

To our knowledge, this is the first study analyzing serum aSMase levels as a biomarker of migraine and a potential therapeutic target for migraine. Importantly, elevated serum levels of aSMase were found in EM and CM patients compared to the control subjects. Furthermore, the decreased SM levels found in both EM and CM indicate that aSMase activity plays a role in migraine. Specifically, a significant decrease in SM and dhSM is observed in serum; however, the minor species are not altered. A possible explanation for this fact lies in the enzymatic activity of aSMase, which has a Km of 0.2 mM [47], a much higher concentration than that of the minor species, some of which are around 0.0002 mM.

As previously mentioned, the molecular mechanisms of migraine are not completely understood; however, several studies have pointed to the implication of inflammation, endothelial dysfunction, and oxidative stress, among others [4,7,48,49,50,51,52].

Therefore, it is worth highlighting that aSMase has been described as a key player in the processes mentioned above. Our present results agree with those and further highlight a key role of this enzyme in the pathogenesis of migraine by aSMase.

According to our findings, previous studies have demonstrated that elevated SM levels were associated with a higher probability of migraine attacks in patients with EM [40]. In this regard, high levels of plasma aSMase, and an increase in SM in plasma, could lead to a greater enzymatic activity that could be translated into an elevated Cer in plasma. Controversially, our study did not show an increase in Cer, but rather a reduction, as reported in previous studies [39,40,41], except for the case of dhCer. Interestingly, Cer, but not dhCer, is a substrate for Cer Kinase (CerK) [53]. CerK phosphorylates Cer, giving rise to Ceramide-1-phosphate (C1P). A previous study described elevated levels of CerK in the plasma of CM patients compared to healthy subjects [54]. Subsequently, increased CerK activity in patients with migraine could explain the low levels of Cer detected despite the increase in aSMase activity both in our and in previous works [39,40,41]. Moreover, C1P has been described as a key regulator of cell and organism homeostasis [55]. C1P is involved in several pathways, such as the mammalian target of rapamycin (mTOR), phosphatidylinositol 3-kinase (PI3K)/Akt, nuclear factor kappa light chain enhancer of activated B cells (NF-κB), protein kinase C-α, c-Jun N-terminal kinase (JNK), vascular endothelial cell growth factor (VEGF), and mitogen-activated protein kinase kinase (MEK)/extracellularly regulated kinases (ERKs) 1/2 [25,56], all described in studies involving migraine. Considering that C1P has been related to processes such as endothelial dysfunction, oxidative stress, vascular tone, and inflammation, among others [55], the potential role of C1P in migraine should be addressed. Additionally, Cer and dhCer can be degraded by ceramidase to give rise to sphingosine and sphinganine, respectively [57,58]. This is an interesting point of view considering that ceramidase activity has been recently linked to the regulation of the inflammatory response [59]. More specifically, it has been determined that acid ceramidase reduces both inflammation and oxidative stress [60].

Oxidative stress arises in response to an excessive accumulation of nitric oxide (NO) and reactive oxygen species (ROS), thus promoting cell damage and activation of pro-inflammatory factors. Oxidative stress is also responsible for the development of endothelial dysfunction [14], therefore playing a potentially crucial role in migraine [7,10,15,16]. In this regard, lipid peroxidation due to an increase in ROS can lead to oxidative degradation of biological membranes, and subsequently to endothelial dysfunction. Recent studies have pointed out that aSMase activation enhances ROS accumulation [61,62,63] and, as a vicious circle, aSMase activity is augmented by reactive oxygen species (ROS) [36,64,65,66]. On the other hand, SM has been described to act as a “biophysical antioxidant” by maintaining the integrity of biological membranes [67].

In turn, endothelial dysfunction promotes the production and stimulation of endothelial NO synthase (eNOS), which increases the production of NO [68]. NO participates in the vasodilation of blood vessels, a process involved in migraine pathogenesis that can trigger migraine headaches [69,70]. Specifically, the interaction of CGRP with the endothelial cells promotes eNOS activation mediated by the adenylyl cyclase (AC)/cAMPK/protein kinase A (PKA) pathway [71]. In this sense, it was observed that NF-κB-dependent iNOS overexpression by a pro-inflammatory stimulus, such as LPS, required the activation of SMase and an elevation in Cer levels in astrocytes, increasing the release of NO [72]. As mentioned, migraine is a neurovascular disorder, including both vascular and neural mechanisms [73]. In this regard, over-excitation of trigeminal neurons promotes the release of factors, such as CGRP or NO, that induce vasodilation and inflammation at the vascular endothelium. Consequent endothelial dysfunction contributes to vascular tone changes that stimulate pro-excitatory factors release by the neurons [49]. Interestingly, several studies have shown that, as observed with CGRP, SMase activation can also promote eNOS-induced vasodilation both in animal models [36] and in humans [74].

As mentioned, over-excitation of trigeminal neurons promotes the release of factors that involve inflammatory processes. It should be noted that aSMase activity can be increased by the action of pro-inflammatory stimuli, such as Tumor Necrosis Factor α (TNF-α), Interleukin-1β (IL-1β) or cytosolic phospholipase A_2_ (cPLA_2_), also involved in migraine [4,5,50,75,76,77].

Altogether, the evidence seems to indicate that aSMase could be a key player in the molecular mechanism of migraine and the endothelial dysfunction observed in certain patients, possibly due to oxidative stress [15,78]. In this sense, FIASMA (Functional Inhibitor of Acid SphingoMyelinAse), a family of drugs with inhibitory effects on aSMase activity, and some of them approved for clinical use as antidepressants for use in humans [79], should be addressed for therapeutic use in migraine.

We must acknowledge some limitations concerning this study, like the fact that future prospective clinical studies with a formal sample size calculation are needed to confirm the results of this pilot study. It should be noted that serum aSMase levels were analyzed, but although a decrease in the concentration of its natural substrate levels was observed, serum aSMase activity should be analyzed in future experiments. Exosome studies in serum have gained great importance due to its involvement in different pathologies, as well as in the homeostasis of the organism [80]. Although most exosomes are composed of phosphatidylcholine (46–89%), there is a small proportion of sphingomyelin and ceramide (2–10%), which may vary depending on physiological conditions [81]. In this sense, the non-separation of exosomes in serum samples can be considered a limitation; however, we understand that according to the concentrations analyzed, interference with exosomes is minimal. Another limitation is the fact that dietary or metabolic variations could influence lipid profiles and enzyme levels. However, we think that this potential bias was mitigated since all blood samples were taken during a fasting period and no differences were found between groups on body mass index.

## 5. Conclusions

In conclusion, elevated serum levels of aSMase were observed in EM and CM patients compared to the control subjects. Furthermore, the decreased SM levels found in both EM and CM indicate that aSMase activity plays a role in migraine. Additional studies should be addressed to elucidate the potential role of aSMase and SM as therapeutic targets for migraine.

## Figures and Tables

**Table 1:** Bivariate analysis by study groups.

Variable	Ctrl N = 23	EM N = 31	CM N = 28	*p*
Age, years				
Ctrl vs. EM	40.2 ± 11.4	41.1 ± 11.6	42.1 ± 10.5	n.s.
Ctrl vs. CM				n.s.
EM vs. CM				n.s.
Females, % (Freq.)	100 (23)	100 (31)	100 (28)	
Ctrl vs. EM				n.s.
Ctrl vs. CM				n.s.
EM vs. CM				n.s.
Time diagnostics (months)	-	16.5 ± 13.3	21.2 ± 13.4	n.s.
EM vs. CM				
Aura, % (Freq.)	-	55 (17)	46 (13)	n.s.
EM vs. CM				
Pain (days/month)	-	8.2 ± 5.4	21.1 ± 4.4	*p* < 0.0001
EM vs. CM				
Visual Analogue Scale (VAS)	-	8.5 ± 1.2	8.6 ± 1.4	n.s.
EM vs. CM				
Treatment, % (Freq.)				
Non-steroidal anti-inflammatories (NSAIDs)	-	87 (27)	82 (23)	n.s.
Triptans	-	48 (15)	54 (15)	n.s.
Beta blockers	-	19 (6)	43 (12)	*p* < 0.05
Calcium antagonists	-	13 (4)	46 (13)	*p* < 0.001
Antidepressants	-	23 (7)	82 (23)	*p* < 0.0001
BOTOX	-	19 (6)	71 (20)	*p* < 0.0001
aSMase (ng/mL)	1.57 ± 0.72	3.62 ± 1.25	3.07 ± 0.96	
Ctrl vs. EM				*p* < 0.0001
Ctrl vs. CM				*p* < 0.0001
EM vs. CM				n.s.

Clinical data represented as mean and standard deviation, and acid sphingomyelinase (aSMase) values, in each studied group. n.s. non-significant.

**Table 2:** Sphingolipidomics data.

Variable	Ctrl	EM	CM	*p*
	N = 21	N = 31	N = 28	
SM Total (pmol equiv/mL)	219,721 ± 36,949	173,534 ± 39,096	158,458 ± 40,009	
Ctrl vs. EM				*p* < 0.01
Ctrl vs. CM				*p* < 0.0001
EM vs. CM				n.s.
SM 14:0 (pmol equiv/mL)	6879 ± 1478	5066 ± 1229	5383 ± 1092	
Ctrl vs. EM				n.s.
Ctrl vs. CM				n.s.
EM vs. CM				n.s.
SM 14:1 (pmol equiv/mL)	918 ± 231	747 ± 252	916 ± 338	
Ctrl vs. EM				n.s.
Ctrl vs. CM				n.s.
EM vs. CM				n.s.
SM 16:0 (pmol equiv/mL)	61,004 ± 14,331	43,457 ± 14,043	38,280 ± 18,334	
Ctrl vs. EM				*p* < 0.0001
Ctrl vs. CM				*p* < 0.0001
EM vs. CM				*p* < 0.01
SM 16:1 (pmol equiv/mL)	10,917 ± 2113	8771 ± 2080	8117 ± 1743	
Ctrl vs. EM				n.s.
Ctrl vs. CM				n.s.
EM vs. CM				n.s.
SM 18:0 (pmol/mL)	15,646 ± 3027	13,317 ± 4120	11612 ± 2842	
Ctrl vs. EM				n.s.
Ctrl vs. CM				*p* < 0.05
EM vs. CM				n.s.
SM 18:1 (pmol equiv/mL)	8784 ± 1985	7240 ± 2125	6803 ± 1716	
Ctrl vs. EM				n.s.
Ctrl vs. CM				n.s.
EM vs. CM				n.s.
SM 20:0 (pmol equiv/mL)	12,533 ± 3107	10,314 ± 2395	11,346 ± 2743	
Ctrl vs. EM				n.s.
Ctrl vs. CM				n.s.
EM vs. CM				n.s.
SM 20:1 (pmol equiv/mL)	6216 ± 1284	4980 ± 1267	4983 ± 1150	
Ctrl vs. EM				n.s.
Ctrl vs. CM				n.s.
EM vs. CM				n.s.
SM 22:0 (pmol equiv/mL)	20,405 ± 5891	15,575 ± 5508	15,365 ± 6447	
Ctrl vs. EM				*p* < 0.01
Ctrl vs. CM				*p* < 0.01
EM vs. CM				n.s.
SM 22:1 (pmol equiv/mL)	12,893 ± 3526	10,229 ± 2599	10,327 ± 3949	
Ctrl vs. EM				n.s.
Ctrl vs. CM				n.s.
EM vs. CM				n.s.
SM 24:0 (pmol/mL)	16,298 ± 3978	12,624 ± 4960	10,669 ± 2997	
Ctrl vs. EM				n.s.
Ctrl vs. CM				*p* < 0.01
EM vs. CM				n.s.
SM 24:1 (pmol/mL)	24,351 ± 4164	22,100 ± 9921	17,234 ± 8593	
Ctrl vs. EM				n.s.
Ctrl vs. CM				*p* < 0.0001
EM vs. CM				*p* < 0.01
SM 24:2 (pmol equiv/mL)	19,424 ± 4164	16,182 ± 4982	14,349 ± 5170	
Ctrl vs. EM				n.s.
Ctrl vs. CM				*p* < 0.01
EM vs. CM				n.s.
SM 24:3 (pmol equiv/mL)	3447 ± 718	2930 ± 634	3075 ± 928	
Ctrl vs. EM				n.s.
Ctrl vs. CM				n.s.
EM vs. CM				n.s.
dhSM (pmol equiv/mL)	8518 ± 1966	7215 ± 2076	7817 ± 2057	
Ctrl vs. EM				n.s.
Ctrl vs. CM				n.s.
EM vs. CM				n.s.
dhSM 14:0 (pmol/mL)	330 ± 146	245 ± 98	303 ± 120	
Ctrl vs. EM				n.s.
Ctrl vs. CM				n.s.
EM vs. CM				n.s.
dhSM 16:0 (pmol equiv/mL)	3872 ± 909	3160 ± 838	3284 ± 739	
Ctrl vs. EM				n.s.
Ctrl vs. CM				*p* < 0.0001
EM vs. CM				*p* < 0.0001
dhSM 18:0 (pmol equiv/mL)	1004 ± 341	842 ± 331	1006 ± 500	
Ctrl vs. EM				n.s.
Ctrl vs. CM				n.s.
EM vs. CM				n.s.
dhSM 20:0 (pmol equiv/mL)	366 ± 155	332 ± 160	416 ± 201	
Ctrl vs. EM				n.s.
Ctrl vs. CM				n.s.
EM vs. CM				n.s.
dhSM 22:0 (pmol/mL)	528 ± 235	470 ± 298	544 ± 296	
Ctrl vs. EM				n.s.
Ctrl vs. CM				n.s.
EM vs. CM				n.s.
dhSM 24:0 (pmol equiv/mL)	140 ± 76	146 ± 89	163 ± 93	
Ctrl vs. EM				n.s.
Ctrl vs. CM				n.s.
EM vs. CM				n.s.
dhSM 24:1 (pmol equiv/mL)	2241 ± 531	2017 ± 547	2099 ± 624	
Ctrl vs. EM				n.s.
Ctrl vs. CM				n.s.
EM vs. CM				n.s.
Cer Total (pmol equiv/mL)	15,213 ± 5718	12,501 ± 5153	14,333 ± 6160	
Ctrl vs. EM				n.s.
Ctrl vs. CM				n.s.
EM vs. CM				n.s.
Cer 14:0 (pmol equiv/mL)	56 ± 27	44 ± 15	62 ± 23	
Ctrl vs. EM				n.s.
Ctrl vs. CM				n.s.
EM vs. CM				n.s.
Cer 16:0 (pmol/mL)	512 ± 180	400 ± 122	465 ± 133	
Ctrl vs. EM				n.s.
Ctrl vs. CM				n.s.
EM vs. CM				n.s.
Cer 16:1 (pmol equiv/mL)	71 ± 18	62 ± 16	69 ± 19	
Ctrl vs. EM				n.s.
Ctrl vs. CM				n.s.
EM vs. CM				n.s.
Cer 18:0 (pmol/mL)	177 ± 54	187 ± 75	195 ± 75	
Ctrl vs. EM				n.s.
Ctrl vs. CM				n.s.
EM vs. CM				n.s.
Cer 18:1 (pmol equiv/mL)	49 ± 13	54 ± 33	50 ± 18	
Ctrl vs. EM				n.s.
Ctrl vs. CM				n.s.
EM vs. CM				n.s.
Cer 20:0 (pmol equiv/mL)	165 ± 50	162 ± 50	178 ± 50	
Ctrl vs. EM				n.s.
Ctrl vs. CM				n.s.
EM vs. CM				n.s.
Cer 20:1 (pmol equiv/mL)	42 ± 13	41 ± 12	45 ± 16	
Ctrl vs. EM				n.s.
Ctrl vs. CM				n.s.
EM vs. CM				n.s.
Cer 22:0 (pmol equiv/mL)	1716 ± 771	1678 ± 515	1658 ± 680	
Ctrl vs. EM				n.s.
Ctrl vs. CM				n.s.
EM vs. CM				n.s.
Cer 22:1 (pmol equiv/mL)	198 ± 61	211 ± 65	202 ± 78	
Ctrl vs. EM				n.s.
Ctrl vs. CM				n.s.
EM vs. CM				n.s.
Cer 24:0 (pmol/mL)	10,409 ± 4950	7813 ± 4690	9395 ± 5560	
Ctrl vs. EM				*p* < 0.0001
Ctrl vs. CM				*p* < 0.05
EM vs. CM				*p* < 0.001
Cer 24:1 (pmol/mL)	1371 ± 504	1369 ± 514	1547 ± 647	
Ctrl vs. EM				n.s.
Ctrl vs. CM				n.s.
EM vs. CM				n.s.
Cer 24:2 (pmol equiv/mL)	445 ± 130	479 ± 164	467 ± 185	
Ctrl vs. EM				n.s.
Ctrl vs. CM				n.s.
EM vs. CM				n.s.
dhCer (pmol equiv/mL)	1991 ± 709	1690 ± 487	2238 ± 833	
Ctrl vs. EM				n.s.
Ctrl vs. CM				n.s.
EM vs. CM				*p* < 0.05
dhCer 16:0 (pmol/mL)	118 ± 85	54 ± 20	136 ± 77	
Ctrl vs. EM				*p* < 0.05
Ctrl vs. CM				n.s.
EM vs. CM				*p* < 0.01
dhCer 18:0 (pmol/mL)	68 ± 66	21 ± 7	102 ± 79	
Ctrl vs. EM				n.s.
Ctrl vs. CM				n.s.
EM vs. CM				*p* < 0.01
dhCer 20:0 (pmol equiv/mL)	21 ± 21	22 ± 7	39 ± 30	
Ctrl vs. EM				n.s.
Ctrl vs. CM				n.s.
EM vs. CM				n.s.
dhCer 22:0 (pmol equiv/mL)	55 ± 14	57 ± 17	58 ± 17	
Ctrl vs. EM				n.s.
Ctrl vs. CM				n.s.
EM vs. CM				n.s.
dhCer 22:1 (pmol equiv/mL)	33 ± 11	34 ± 12	33 ± 12	
Ctrl vs. EM				n.s.
Ctrl vs. CM				n.s.
EM vs. CM				n.s.
dhCer 24:0 (pmol/mL)	329 ± 158	334 ± 145	399 ± 205	
Ctrl vs. EM				n.s.
Ctrl vs. CM				*p* < 0.05
EM vs. CM				*p* < 0.05
dhCer 24:1 (pmol/mL)	296 ± 92	296 ± 104	381 ± 172	
Ctrl vs. EM				n.s.
Ctrl vs. CM				*p* < 0.01
EM vs. CM				*p* < 0.001

Sphingolimidomic data are presented as the mean and standard deviation. Since standards for all identified lipids were not available, the total amounts of lipids are given as pmol equivalents/mL (pmol equiv/mL) relative to each specific standard (N-dodecanoylsphingosine for Cer and dhCer and N-dodecanoylsphingosylphosphorylcholine for SM and dhSM). On the other hand, values are in pmol/mL if natural sphingolipids standards were available (see Section 2). n.s. non-significant.

## Data Availability

The authors confirm that the data supporting the findings of this study are available within this article. Raw data supporting this study’s findings are available from the corresponding author upon reasonable request.

## Supplementary Materials

The following supporting information can be downloaded at: https://www.mdpi.com/article/10.3390/antiox14020159/s1, Figure S1: Sphingolipid analysis were carried out in an Acquity ultra-high-performance liquid chromatography (UHPLC) system (Waters, USA) connected to a Time of Flight (LCT Premier XE).

## Abbreviations

The following abbreviations are used in this manuscript:

aSMase	Acid sphingomyelinase
BBB	Blood–brain barrier
C1P	Ceramide 1-phosphate
Cer	Ceramide
CGRP	Calcitonin-gene related peptide
CM	Chronic migraine
cPLA_2_	Cytosolic phospholipase A_2_
dhCer	Dihydro-ceramide
dhSM	Dihydro-sphingomyelin
EM	Episodic migraine
FIASMA	Functional Inhibitor of Acid SphingoMyelinAse
IL-1β	Interleukin-1β
NO	Nitric oxide
NOS	Nitric oxide synthase
ROS	Reactive oxygen species
TNF-α	Tumor Necrosis Factor α
