# Supplementary material for: Elevated Serum Levels of Acid Sphingomyelinase in Female Patients with Episodic and Chronic Migraine
Source: Antioxidants (Basel). 2025 Jan 29;14(2):159. doi: 10.3390/antiox14020159 (PMC11851676; doi:10.3390/antiox14020159)
Supplement: Supplementary file 1 [file antioxidants-14-00159-s001.zip › antioxidants-3412083-supplementary.pdf]

Supplementary Figures

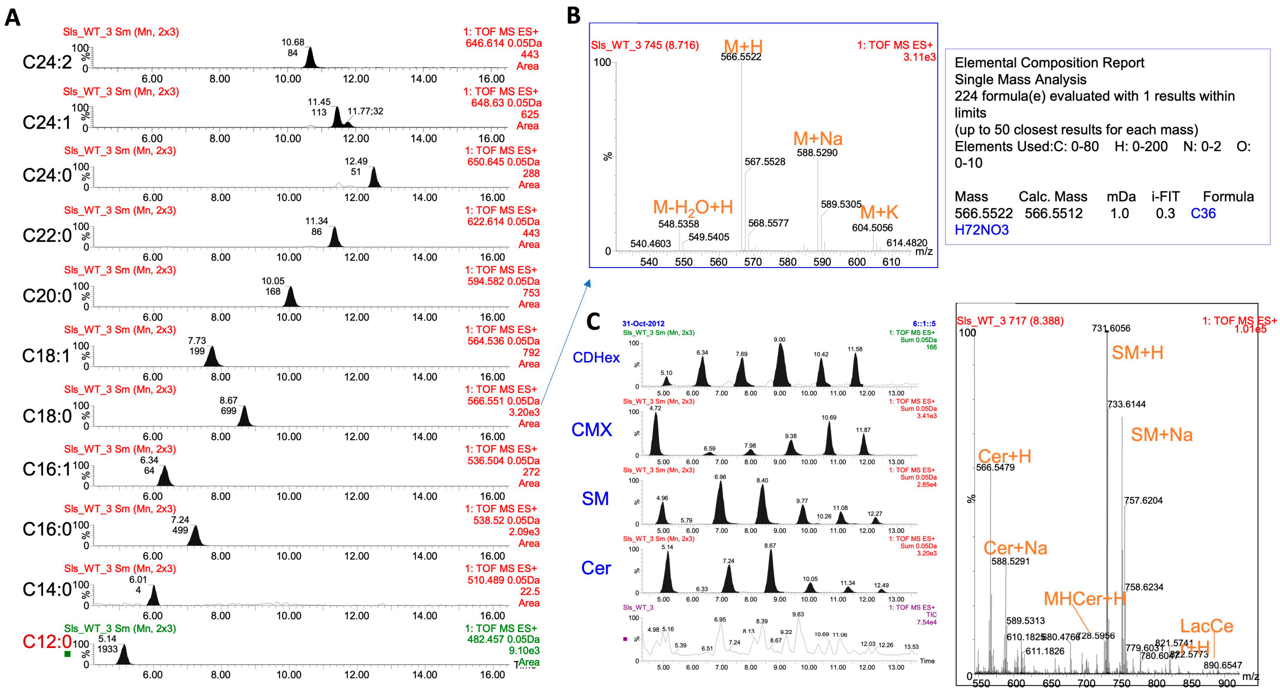

Figure S1. Sphingolipid analysis were carried out in an Acquity ultra-high-performance liquid chromatography (UHPLC) system (Waters, USA) connected to a Time of Flight (LCT Premier XE). A) Selected ion chromatograms of molecular ions of Cer-C12:0 to Cer-C24:2. B) A representative spectrum for C18:0-Cer eluting at 7.8 min, and the elemental composition of the [M+1]+ ion (m/z 566.5522), which value differs 1mDa from the theoretical one, are shown. C) From bottom to top, TIC and combined selected ion chromatograms from Cers, SMs, CerMXs (CMX) and CerDXs CDHex) are shown. Spectrum at 8.4 min displaying m/z values of sphingolipids containing a C18:0 acyl chain.
